# c-MYB is a transcriptional regulator of *ESPL1*/Separase in *BCR-ABL-*positive chronic myeloid leukemia

**DOI:** 10.1186/s40364-016-0059-2

**Published:** 2016-03-02

**Authors:** Wiltrud Prinzhorn, Michael Stehle, Helga Kleiner, Sabrina Ruppenthal, Martin C. Müller, Wolf-Karsten Hofmann, Alice Fabarius, Wolfgang Seifarth

**Affiliations:** III. Medizinische Klinik (Hämatologie und Onkologie), Wissenschaftliches Labor, Medizinische Fakultät Mannheim der Universität Heidelberg, Pettenkofer Str. 22, 68169 Mannheim, Germany

**Keywords:** *ESPL1*/Separase, c-MYB, CML, Imatinib, BCR-ABL, Genomic stability

## Abstract

**Background:**

Genomic instability and clonal evolution are hallmarks of progressing chronic myeloid leukemia (CML). Recently, we have shown that clonal evolution and blast crisis correlate with altered expression and activity of Separase, a cysteine endopeptidase that is a mitotic key player in chromosomal segregation and centriole duplication. Hyperactivation of Separase in human hematopoietic cells has been linked to a feedback mechanism that posttranslationally stimulates Separase proteolytic activity after imatinib therapy-induced reduction of Separase protein levels.

**Methods and Results:**

In search for potential therapy-responsive transcriptional mechanisms we have investigated the role of the transcription factor c-MYB for Separase expression in CML cell lines (LAMA-84, K562, BV-173) and in clinical samples. Quantitative RT-PCR and Western blot immunostaining experiments revealed that c-MYB expression levels are decreased in an imatinib-dependent manner and positively correlate with Separase expression levels in cell lines and in clinical CML samples. RNA silencing of c-MYB expression in CML cell lines resulted in reduced Separase protein levels. Gelshift and ChIP assays confirmed that c-MYB binds to a putative c-MYB binding sequence located within the ESPL1 promoter.

**Conclusions:**

Our data suggest that *ESPL1*/Separase is a regulatory target of c-MYB. Therefore, c-MYB, known to be required for BCR-ABL-dependent transformation of hematopoietic progenitors and leukemogenesis, may also control the Separase-dependent fidelity of mitotic chromosomal segregation and centriole duplication essential for maintenance of genomic stability.

## Background

*ESPL1*/Separase, a cysteine endopeptidase, is a key player of chromosomal segregation and centrosome duplication. In mitotic anaphase, it accomplishes proteolytic cleavage of Cohesin, a “glue” multi-protein complex that is responsible for cohesion of sister chromatids and of mother and daughter centrioles, the perpendicular oriented core structures of centrosomes [1–3]. Proper temporal and spatial activation of Separase proteolytic activity warrants chromosomal fidelity by establishing an accurate chromosomal segregation [4]. Furthermore, it is an essential prerequisite for semiconservative centriole duplication as the disengagement of mother and daughter centrioles licenses the cell cycle-associated duplication of centrosomes [5]. Failure to do so will result in premature segregation of chromatids and/or formation of anaphase bridges from lagging chromosomes [6]. Moreover, unscheduled (cell cycle uncoupled) activation of Separase can lead to aberrant high numbers of centrosomes (i.e. centrosome amplification) and subsequently to a defective mitotic spindle apparatus [7]. Both defects cause the emergence of aberrant karyotypes (aneuploidy), a hallmark of most advanced human malignancies [8–10]. In non-malignant cells where chromosome segregation and centrosomal duplication are tightly coupled to the cell cycle, Separase is temporary activated on metaphase to anaphase transition [11, 12]. Separase is tightly regulated on both translational and posttranslational levels. The latter includes multiple inhibitory mechanisms combining Securin binding, specific serine residue phosphorylation (pSer1126) by CyclinB1/CDK1, autocatalytic cleavage, and PP2A-dependent stabilization of Separase-bound Securin. All these mechanisms work together to prevent ectopic and unscheduled activation of intracellular Separase molecules [13–16].

In human cancer, *ESPL1*/Separase is frequently overexpressed and the resulting deregulated proteolytic activity is associated with the occurrence of supernumerary centrosomes, chromosomal missegregation and aneuploidy [6, 9, 13, 17]. Overexpression of Separase in the mammary gland of a MMTV-*ESPL1* mouse model led to the development of highly aneuploid mammary carcinomas with high levels of chromosomal instability and aggressive disease phenotypes [18]. Consequently, Separase has been identified as an aneuploidy promoter that, when overexpressed and hyperactive, functions as an oncogene and renders cells susceptible not only for chromosomal missegregation-induced aneuploidy but also for DNA damage and loss of key tumor suppressor gene loci associated with tumorigenesis and disease progression [18–20].

This holds true for chronic myeloid leukemia (CML) as well, a clonal neoplastic disorder of hematopoietic stem cells caused by the genomic reciprocal translocation t(9;22)(q34;q11), which results in the formation of the Philadelphia chromosome. The fusion product, a BCR-ABL tyrosine kinase (TK) with deregulated TK activity, is the key player in CML pathogenesis. It affects various downstream signaling pathways by reprogramming the prior lineage commitment of hematopoietic stem and early progenitor cells [21]. Compromising multiple aspects of cellular behavior, including proliferation, apoptosis, cell to cell signaling and differentiation, the BCR-ABL oncoprotein triggers aberrant clonal hematopoiesis and drives disease progression from chronic phase (CP) toward the fully transformed phenotype of blast crisis (BC) [22]. Imatinib (IM) is a selective TK inhibitor (TKI) and presents one of the current first line treatments for CML [23, 24]. Despite significant decreases in BCR-ABL mRNA levels in the bone marrow compartment under IM long-term therapy, persistence of residual CML clones with low BCR-ABL expression and insensitivity to IM treatment makes disease eradication by TKI treatment unlikely [13, 25, 26]. Recent evidence suggests that kinase activity of BCR-ABL oncoprotein in CML stem cells is inhibited by TKI treatment without affecting CML stem cell survival [27, 28]. Obviously, additional cellular mechanisms promote CML stem cell survival and maintenance, rendering these cells TKI resistant and eventually promote relapse [29, 30]. About 35 % of patients in CP develop resistance or intolerance to IM and frequently undergo clonal evolution [31]. Clonal evolution denotes a heterogeneous entity of clonal molecular changes in BCR-ABL-positive hematopoietic stem/progenitor cells and has been described in about 30 % and 80 % of patients in accelerated phase (AP) and BC, respectively [32]. Emergence of altered chromosome numbers, collectively termed aneuploidy, involves an additional derivative chromosome 22, chromosome 17 abnormalities, trisomy 8, and are associated with poor prognosis [33, 34].

Recently, we have reported that enhanced rates of acquired chromosomal aberrations, clonal evolution and fast disease progression (time to BC) in CML patients undergoing long-term IM treatment correlate with enhanced proteolytic activity of Separase in the respective *in vitro* models [35]. Mechanistically, this was linked to a BCR-ABL-dependent regulatory feedback mechanism that posttranslationally stimulates Separase proteolytic activity after IM-induced decreases of Separase expression in b3a2 BCR-ABL fusion type CML cell lines [35]. To date, it was unclear what underlying transcriptional or translational mechanism may be involved in the IM-dependent regulation of Separase expression in BCR-ABL-positive cells.

The transcription factor c-MYB is known to play an important role in BCR-ABL-dependent leukemogenesis. The expression of c-MYB is enhanced by BCR-ABL [36–38]. C-MYB is a 75 KDa nuclear protein and a leucine zipper transcription factor encoded by the proto-oncogene *c-MYB*. It plays a pivotal role in proliferation, survival and differentiation of normal myeloid progenitors [39]. Conditional knockout of *c-MYB* expression in adult hematopoietic stem cells causes loss of self-renewal due to impaired proliferation and accelerated differentiation [40]. On the other hand, overexpression of *c-MYB* in myeloid and erythroid cell lines has been demonstrated to block differentiation and prevents maturation-associated growth arrest [41]. Aberrant (enhanced) *c-MYB* expression has been found in various human malignancies including T-cell leukemia and acute and chronic myeloid leukemias [42]. Moreover, various genetic lesions affecting c-MYB activity in human leukemias, such as chromosomal translocation, gene duplication and truncations have been reported [39, 43, 44]. However, in CML cells, the *c-MYB* gene has been reported to be intact but protein levels are often increased, in part, due to enhanced protein stability via BCR-ABL-regulated activation of PI-3 K/Akt/GSKIIIß dependent pathways [36]. This altered regulatory mechanism has been considered to explain why leukemic blast cells appear to depend on high c-MYB expression levels more than their normal counterparts [45]. Functional studies of c-MYB by ChIP-Seq experiments revealed that c-MYB functions as a hematopoietic master regulator [46]. It binds directly near or within 793 genes thereby affecting more than 2300 genes that make up the gene signatures for normal and leukemic stem/progenitor cells and myeloid development. Despite being usually considered as a transactivator, c-MYB is also able to directly repress many target genes pointing to an important role for myelopoiesis and leukemogenesis through both positive and negative transcriptional regulation [47]. For example, c-MYB modulates the expression of *CD34, c-Kit, c-Myc, Flt-3 and Bcl-2* all playing important roles for proliferation and survival of hematopoietic cells [38]. Moreover, c-MYB directly regulates CyclinB1 expression and contributes to the control of the G2/M cell cycle phase [48, 49], thereby directly controlling one of the key posttranslational inhibitors of Separase [13, 14].

In this study, we set out to investigate the role of c-MYB for the regulation of *ESPL1*/Separase expression in CML. We report that c-MYB binds to a c-MYB binding motif located within the *ESPL1* promoter and functions as a positive regulator of *ESPL1*/Separase expression as demonstrated by *c-MYB*-directed siRNA silencing. Moreover, IM treatment led to equally decreased c-MYB and *ESPL1*/Separase expression levels in CML cell lines and primary cells.

## Results

In search for transcriptional mechanisms that could explain the IM-associated downregulation of Separase protein levels we analyzed the conditional context between c-MYB expression, Separase and IM treatment. Confirming and expanding previously published work we performed cell culture experiments on four human cell lines [13, 35]. Of these, U937 cells served as model for leukemic but BCR-ABL-negative cells. All cell lines were treated with therapeutic doses of IM (1 to 5 μM) as performed in our previous studies [13, 50–52]. In accordance with data from extensive studies on the dose-dependent effects and time kinetics of IM we applied lower IM doses (range: 1 to 2.5 μM) for leukemia-derived p210BCR-ABL-positive cells (LAMA-84 and K562) than for p210BCR-ABL-negative cells (U937, 5 μM) [53, 54]. Treating CML cell lines with IM doses higher than 2.5 μM for a longer period than 24 h impeded the collection of sufficient viable cells for Western blot analysis (data not shown).

### Concerted decrease of c-MYB and ESPL1 expression levels under IM treatment

**Fig. 1:**
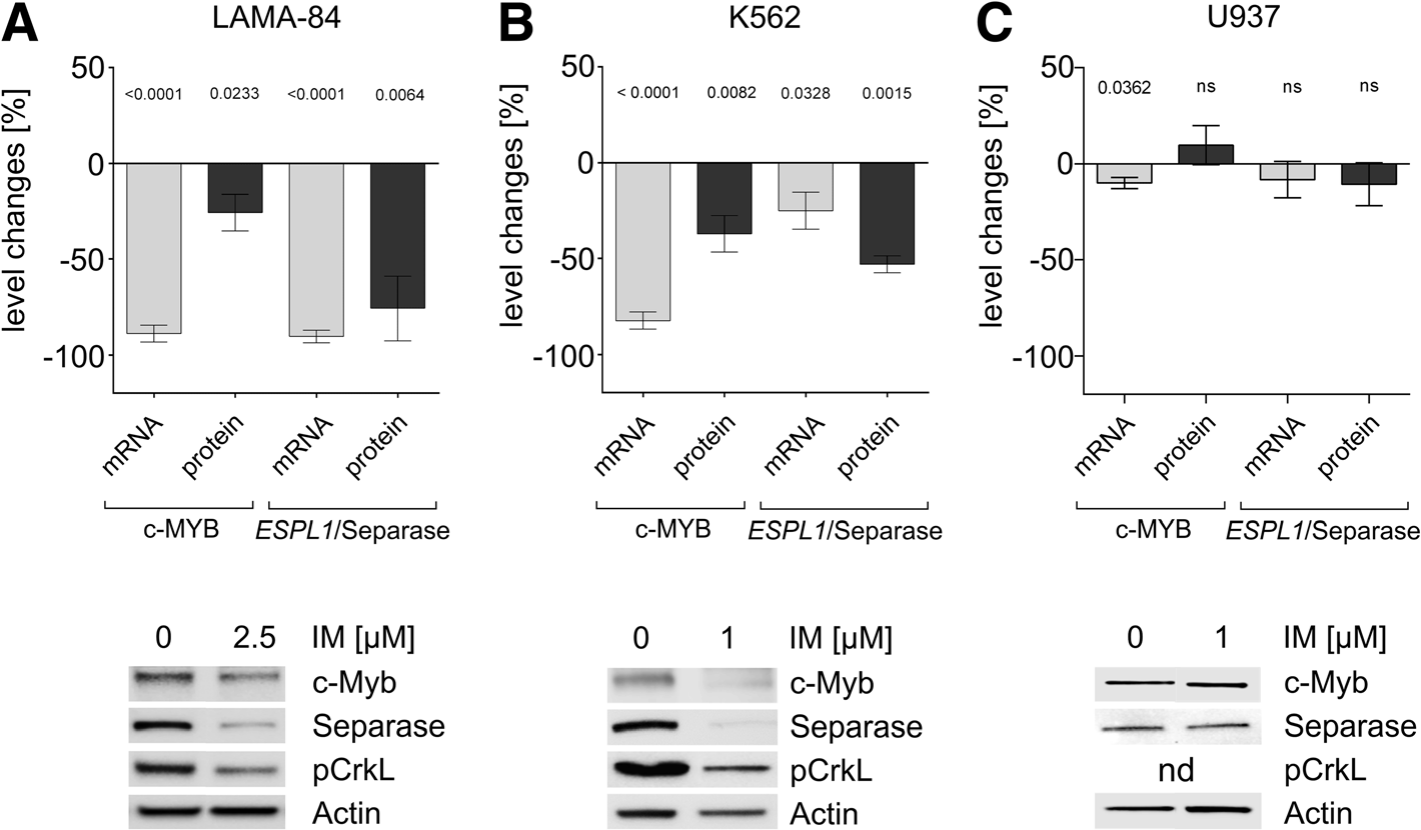
Analysis of *c-MYB* and *ESPL1* transcript levels, c-MYB and Separase protein levels in LAMA-84 (**a**), K562 (**b**) and BCR-ABL-negative control cells (U937) (**c**) upon IM treatment. Treatment (dose, period) was performed as noted in Table 1. Level changes (Δ-values in %) are shown as calculated from comparison with the corresponding untreated cells. Upper panel. Transcript levels were analyzed by qRT-PCR, protein levels by Western blot immunostaining densitometry. Representative Western blot images are shown in the lower panel. In all qRT-PCR experiments the housekeeping gene *Gus* (beta-glucuronidase) served as internal standard. For Western blot immunostaining experiments Actin was used as loading control and reference parameter. Densitometric data are derived from at least triplicate experiments and are denoted in Table 1. P-values are given above the respective column. Abbreviations: ns, not significant; nd, not determined

Treatment of the CML cell lines LAMA-84 and K562 with therapeutic IM doses for 24 h revealed decreased expression levels for c-MYB and *ESPL1*/Separase on both transcriptional and protein levels as shown on representative Western blot composite images (Fig. 1, Table 1). It should be emphasized that the weak treatment schedule (≤2.5 μM IM for 24 h) still enabled CrkL phosphorylation (Fig. 1, lower panel). Thus, when compared to the respective untreated cells, the decreasing c-MYB protein levels in LAMA-84 (−25.7 ± 9.6 %) and K562 (−37.1 ± 9.6 %) cells concurred with decreased *ESPL1* transcript levels of −90 ± 3.3 % and −25.0 ± 9.7 %, respectively. One might argue that the observed decline may be due to IM-related changes in the cell cycle. However, FACS analysis of tested cells revealed no differences neither in G2/M cell proportion nor in the apoptotic cell fraction (<12 %) that could clarify the observed decreases in c-MYB and *ESPL1*/Separase expression levels (compare Fig. 3b in Ref. [13]). Treatment of BCR-ABL-negative control cells (U937) with IM revealed no changes in c-MYB protein and *ESPL1*/Separase expression levels (Table 1, Fig. 1).

**Table 1:** Percent changes (Δ-values are differences between means) in *c-MYB* and *ESPL1*/Separase transcript and protein levels under IM treatment when compared to corresponding untreated cells

Cell line, dose and period of treatment	*c-MYB* transcript levels^a^	c-MYB protein levels^b^	*ESPL1* transcript levels^a^	Separase protein levels^b^
LAMA-84, 2.5 μM IM, 24 h	−88.8 ± 4.4, *p* < 0.0001	−25.7 ± 9.6, *p* = 0.0233	−90.3 ± 3.3, *p* < 0.0001	−75.8 ± 16.8, *p* = 0.0064
K562, 1 μM IM, 24 h	−82.4 ± 4.4, *p* < 0.0001	−37.1 ± 9.6, *p* = 0.0082	−25.0 ± 9.7, *p* = 0.0328	−53.1 ± 4.4, *p* = 0.0015
U937, 5 μM IM, 48 h	−9.1 ± 2.9, *p* = 0.0363	+9.8 ± 10.2, *p* = 0.3942	−8.1 ± 9.5, *p* = 0.4198	−10.5 ± 11.2, *p* = 0.3775

^a^ Δ-values [%] were calculated from at least triplicate qRT-PCR experiments and were normalized to the housekeeping gene *Gus*

^b^ Δ-values [%] are derived from at least triplicate Western blot immunostaining experiments. All protein values were normalized to Actin as loading control

*Abbreviations*: *d* days, *h* hours, *IM* imatinib; + increase, − decrease

**Fig. 2:**
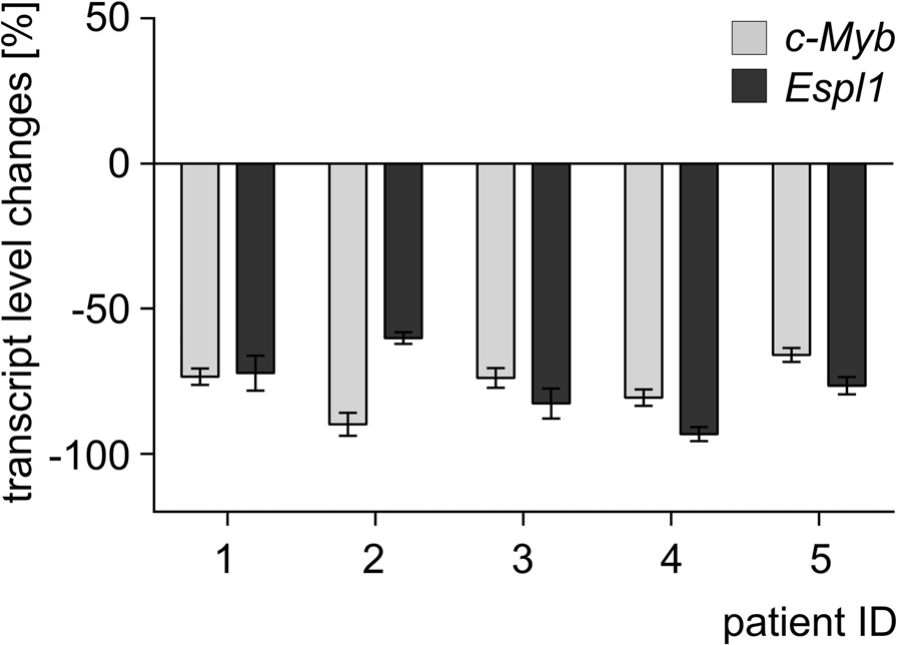
Comparative analysis of *c-MYB* and *ESPL1* transcript levels in paired cDNA samples of CML patients (*n* = 5) before and under IM therapy. Percent changes (Δ-values are differences between means) are shown corresponding to expression level changes within paired samples, each pair derived from the same patient at differing time points (sample at diagnosis (before IM treatment) vs. sample after major molecular response (MMR) achievement under IM therapy). All transcript levels were normalized to *Gus* and represent mean values of triplicate qRT-PCR assays

Similar results were obtained when paired clinical samples (*n* = 5), each pair derived from the same CML patient before (at diagnosis) and during IM therapy were comparatively monitored for *c-MYB* and *ESPL1* transcript levels (Fig. 2). Two female and three male patients, all with b3a2 *BCR-ABL* fusion type, were analyzed. The median age was 58 years (range, 47 to 78). Mean time between diagnosis and sampling of the second specimen from the same patient after achievement of MMR under IM treatment was 3.6 years (range, 2 to 6.5). These experiments suggest synchronous regulatory mechanisms for c-MYB and *ESPL1*/Separase expression upon IM treatment *in vitro* and *in vivo*.

### Decreased Separase expression levels after c-*MYB* silencing in CML cell lines

**Fig. 3:**
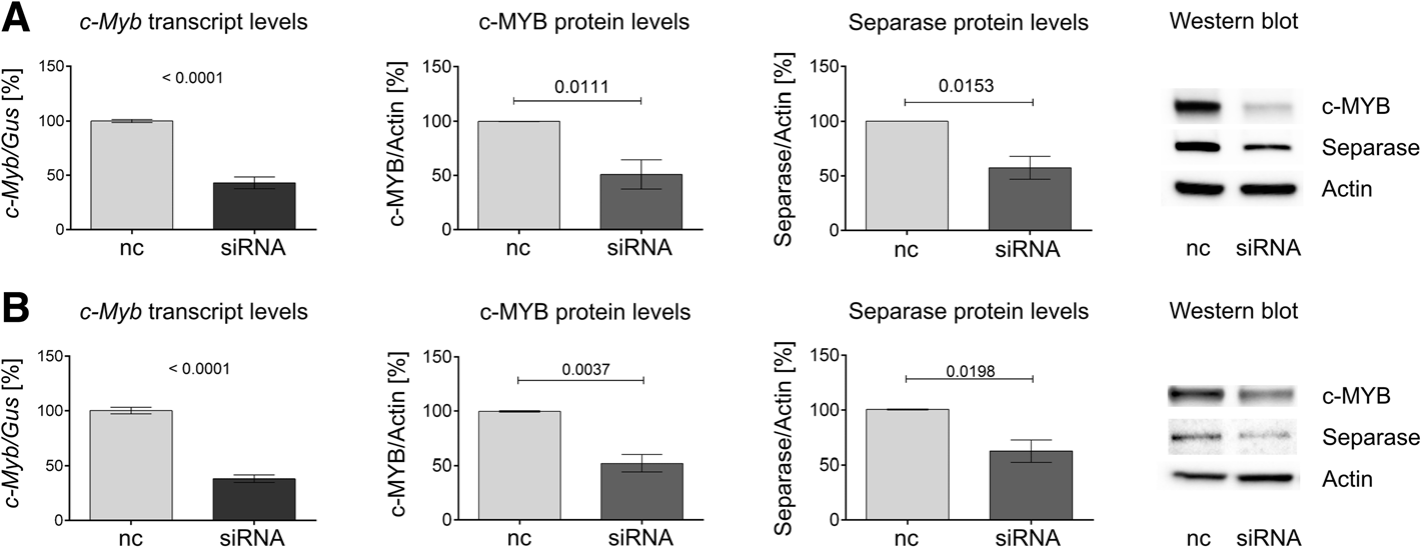
Separase expression after *c-MYB* silencing by RNAi in BV-173 and LAMA-84 cells. BV-173 (panel **a**) and LAMA-84 cells (panel **b**) were treated with negative control siRNA (nc) and *c-MYB*-specific siRNA (siRNA). *C-MYB* transcript levels are measured by qRT-PCR (left column). The house-keeping gene *Gus* (beta-glucuronidase) served as internal standard. C-MYB and Separase regulation on protein levels were determined by quantitative Western blot immunostaining experiments 48 h post transfection (middle and right columns, respectively). Corresponding representative Western blot images are shown in the very right panels of A and B. Actin served as loading control for Western blot immunostaining. All densitometric data are derived from at least triplicate experiments

To test the direct regulatory influence of c-MYB on Separase expression we silenced *c-Myb* transcription in LAMA-84 and BV-173 cells and monitored the influence of the resulting decline in c-MYB protein levels on Separase expression (Fig. 3). The siRNA-induced decrease in available c-MYB molecules resulted in decreased c-MYB protein levels in BV-173 (−49 ± 13.6 %) and LAMA-84 cells (−47.6 ± 7.9 %). This suggests a direct regulatory relationship between c-MYB and *ESPL1*/Separase.

### c-MYB binds to a putative c-MYB recognition site located within the *ESPL1* promoter

**Fig. 4:**
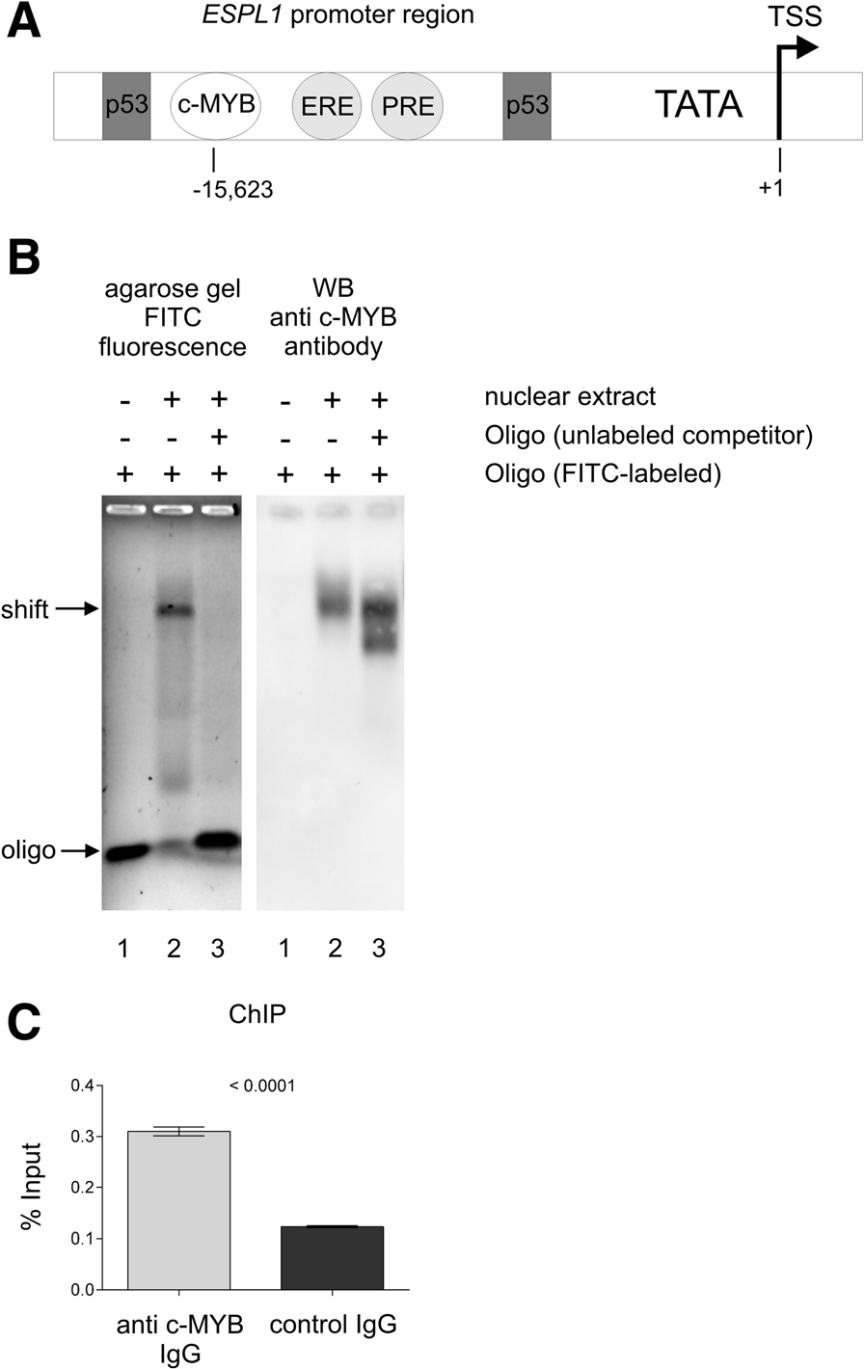
Electrophoretic mobility shift assay (EMSA) using synthetic *ESPL1* promoter-derived c-MYB binding site probes and Chromatin immunoprecipitation (ChIP). **a** Schematic drawing depicting the *ESPL1* Separase promoter and location of predicted regulatory DNA motifs (drawing not to scale) (Pati 2008). The arrow shows the predicted transcription start site (TSS). Abbreviations: TATA, TATA box; PRE, progesterone responsive element; ERE, estrogen responsive element; p53, p53 binding element; c-MYB, predicted c-MYB binding element. Numbers denote upstream distances with respect to the TSS. **b** A FITC-labeled double stranded DNA oligonucleotide corresponding to the putative c-MYB binding site of the *ESPL1* promoter was incubated with native BV-173 nuclear extract. DNA/protein complexes were resolved on a 0.5 % TBE 1.0 % native LE/GTG agarose gel. The left panel (tonal inversion) shows FITC-related fluorescence signaling of the gel before blotting, the right panel depicts the corresponding anti-c-MYB Western blot immunostaining. The lanes represent: lane 1, DNA target (FITC-labeled oligonucleotide) without nuclear extract; lane 2, DNA target with nuclear extract; lane 3, DNA target with nuclear extract and with 100fold molar excess of analogous unlabeled oligonucleotide as binding competitor. **c** ChIP analysis of BV-173 cells. DNA fragments immunoprecipitated by anti-c-MYB IgG and a non-binding control IgG were amplified by qRT-PCR. Results are expressed as percentage of input (average). ChIP results are derived from at least triplicate qRT-PCR measurements

The *ESPL1* promoter features two hormone responsive elements and two TP53 binding sites that are located 12,777 and 16,059 bases upstream of the predicted transcription start site (TSS) [20]. For details go to the Human Genome Browser (http://www.ncbi.nlm.nih.gov/gene?cmd=Retrieve&dopt=Graphics&list_uids=9700) and find the TSS at position 53,662,083 (+ strand) of the NC_000012.11 Chromosome 12 Reference GRCh37.913 Primary Assembly. The Champion ChIP Transcription Factor Search Portal based on SABiosciences’ proprietary database known as DECODE (DECipherment Of DNA Elements) was used to identify the putative c-MYB binding site within the *Espl1* promoter (for more information see http://www.sabiosciences.com/chipqpcrsearch.php?app=TFBS). A hypothetical c-MYB binding site was found 15,623 bases upstream of the TSS (53,646,460 - 53,646,470) and thus, is located between both TP53 sites (see Fig. 4a). In order to determine whether the putative c-MYB binding site of the *ESPL1* promoter can actually bind the c-MYB protein, we performed electrophoretic mobility shift assays (EMSA). Native nuclear extracts prepared from exponentially growing BV-173 cells were incubated with a FITC-labeled double stranded oligonucleotide (Fig. 4b lane 1) featuring the c-MYB binding site of the *ESPL1* promoter. As shown in Fig. 4b left panel, addition of nuclear extract to the FITC-labeled oligonucleotide (lane 2) led to the formation of an oligo/protein complex with retarded electrophoretic migration (shift). Addition to the incubation mix of a 100fold molar excess of unlabeled c-MYB binding site oligonucleotide successfully competed with the formation of the shifted signal (lane 3). For corroboration of c-MYB protein binding, the native agarose gel was blotted under denaturing conditions followed by immunostaining with an anti-c-MYB antibody (right panel). The contribution of c-MYB to the shift signal (lane 2) was confirmed. In addition, the unlabeled competitor oligonucleotide (lane 3) appeared as shifted signal as well confirming specific abrogation of the FITC signal (left panel, lane 3) by the unlabeled competitor DNA. The second and lower band may be due altered electrophoretic mobility of at least some of the native oligo/c-MYB complexes after addition of 100fold excess of the unlabelled competitor. To demonstrate *in vivo* binding of c-MYB protein to the putative c-MYB recognition site located within the *ESPL1* promoter we performed ChIP experiments on BV-173 cells. As shown in Fig. 4c a significant enrichment of ESPL1 promoter-related target DNA was observed when using a c-MYB-specific IgG antibody for chromatin immunoprecipitation when compared to the unspecific IgG antibody control. In summary, our *in vitro* and *in vivo* data suggest that c-MYB directly binds to the *ESPL1* promoter and positively regulates *ESPL1*/Separase expression.

## Discussion

In search for transcriptional mechanisms that may explain the previously observed IM-associated downregulation of Separase protein levels in CML [13] followed by posttranslational hyperactivation of Separase proteolytic activity, we have analyzed the conditional context between c-MYB expression, Separase and IM treatment. We found that the transcription factor c-MYB, known to play a pivotal role in proliferation, survival and differentiation of normal myeloid progenitors, is a direct positive regulator of Separase expression. Specifically, we demonstrated by EMSA (Fig. 4b) and ChIP that c-MYB interacts with a putative c-MYB binding motif located within the *ESPL1* promoter. In fact, in all tested CML cell lines (K562, LAMA-84, BV-173) and in primary cells from CML patients coinciding c-MYB and *ESPL1*/Separase expression levels irrespective to therapeutic treatment (IM) and silencing (siRNA) conditions (Figs. 1, 2 and 3) were observed. This suggests that the *ESPL1* promoter is under transcriptional control of the transcription factor c-MYB concurring with former results of Kohmura and coworkers who found a downregulation of c-MYB mRNA levels in K562 cells after incubation with IM [55].

Our results further coincide with previous studies reporting that p210BCR-ABL functions as enhancer of c-MYB expression and plays an important role in BCR-ABL-dependent leukemogenesis as the leukemic blast cells appear to rely on high levels of c-MYB protein more than normal progenitors [37, 45]. Moreover, c-MYB promotes centriole duplication and, when mutated, can lead to centrosome amplification as demonstrated in mouse and Drosophila model systems [56, 57]. Previous microarray data point to c-MYB as part of the regulatory network associated with CML progression including clonal evolution and genetic instability [58, 59].

The positive regulatory impact of c-MYB on *ESPL1*/Separase expression explains well the observations of Patel and Gordon who found abnormally high Separase expression levels in CML cells of chronic and blast phase when compared to normal CD34+ cells. It was then speculated that BCR-ABL expression may upregulate *ESPL1*/Separase and influence the occurrence of centrosomal aberrations [60].

Our data provide the first proof of direct regulatory relationship between BCR-ABL, c-MYB and *ESPL1*/Separase and give a plausible mechanistic explanation on how BCR-ABL may trigger dislocation between the centrosome-centriole cycle and the cell cycle in CML contributing to clonal evolution and genomic instability. The therapeutic administration of IM “normalizes” c-MYB protein levels - as shown in LAMA-84 and K562 cells (Fig. 1) - either by slowing down the transcriptional rate of *c-MYB* or by antagonizing enhanced c-MYB protein stability elicited by BCR-ABL via the PI-3 K/AKT pathway [36, 55]. The anticipated decrease of c-MYB protein levels after IM treatment is also in line with data of Flamant and coworkers who demonstrated that the drug results in a rapid increase in the expression of regulatory microRNAs such as miR-150 that is a strong negative regulator of c-MYB protein expression [61, 62].

## Conclusions

In conclusion, our data suggest that *ESPL1*/Separase is a regulatory target of c-MYB and that c-MYB expression is modulated by IM treatment. Therefore, c-MYB, known to be required for BCR-ABL-dependent transformation of hematopoietic progenitors and leukemogenesis, may also influence Separase-related proteolytic events such as chromosomal segregation and centriole duplication, the ordered course of which is essential for maintenance of centrosomal and genomic stability.

## Methods

### Cell lines and culture conditions

Four human cell lines (K562, LAMA-84, BV-173, U937) were investigated. Of these, K562, LAMA-84 and BV-173 are BCR-ABL-positive IM responsive CML cell lines. U937 cells served as BCR-ABL-negative control cells. All cell lines were obtained from the DSMZ (German Collection of Microorganisms and Cell Cultures, Braunschweig, Germany) and were cultured in RPMI-1640 medium supplemented with 10 % fetal bovine serum and 1 % penicillin-streptomycin (Gibco/Invitrogen, Karlsruhe, Germany) at 37 °C in 5 % CO_2_ atmosphere. Exponentially growing cells were used in at least triplicate experiments.

### Patients and ethics statement

Paired cDNA samples of randomly chosen CML patients (*n* = 5) from the German CML-Study IV (registered at www.clinicaltrials.gov as # NCT00055874) were investigated by qRT-PCR. Each sample pair was derived from peripheral blood of the same patient at the time point of diagnosis (before IM treatment) and after achievement of major molecular response (MMR) under IM therapy. Blood sampling was performed in the context of regular therapeutic monitoring. The procedure followed the declaration of Helsinki and was approved by the IRB/Medizinische Ethikkommision II der Medizinischen Fakultät Mannheim der Ruprecht-Karls-Universität Heidelberg. (http://www.umm.uni-heidelberg.de/inst/ethikkommission, # 2013-509 N-MA from 2013-02-21). Written informed consent was obtained from all patients. Two female and three male patients, all with b3a2 *BCR-ABL* fusion type, were analyzed. The median age was 58 years (range, 47 to 78). Mean time between diagnosis and sampling of the second specimen from the same patient after achievement of MMR under IM treatment was 3.6 years (range, 2 to 6.5).

### IM treatment

Cells were treated with IM (Biomol GmbH, Hamburg, Germany) in concentrations of 1 to 5 μM for 24 h (K562, LAMA-84) and 48 h (U937) according to the rationale pointed out in references [13, 35]. Untreated cells served as controls.

### Western blot analysis, antibodies

Western blot immunostaining of Separase and Actin was performed as described previously [13]. For c-MYB detection, an anti-c-MYB monoclonal rabbit antibody (ab45150; Abcam, Cambridge, UK) at a dilution of 1:10,000 was used. Signals were visualized with a ChemiDoc™ XRS+ System (BIO-RAD, München, Germany) after secondary antibody staining (goat anti-rabbit IgG HRP conjugated antibody (1:10,000; Santa Cruz Biotechnology Inc., Heidelberg, Germany) utilizing SuperSignal West Femto Maximum Sensitivity Substrate (Thermo Fisher Scientific, Bonn, Germany). Image acquisition and densitometric analysis was performed using Image Lab Software (version 3.0.1, BIO-RAD). All values were normalized with Actin as loading control. Image cropping and tonal adjustments across the entire image were performed with Adobe Photoshop CS4 (Adobe Systems Inc., San Jose, CA, USA).

### RNA extraction and quantification of *ESPL1* and *c-MYB* transcripts by qRT-PCR

Total RNA was extracted using RNeasy kit (Qiagen, Hilden, Germany) and reverse transcribed using Superscript II kit (Gibco/Invitrogen). For quantification of *ESPL1* and *c-MYB* transcript levels, the commercial Hs_MYB_1_SG and Hs_ESPL1_1_SG QuantiTect Primer Assays (Qiagen) were employed according to the instructions (two-step Light Cycler 480 protocol) of the manufacturer, respectively. For normalization, the housekeeping gene beta-glucuronidase (*Gus*, NM_000181, GUSB, primer set Hs_GUSB_1_SG, QuantiTect Primer Assay, Qiagen) was amplified. QRT-PCR was performed with the Roche LightCycler 480 System, using LC480 DNA Master SYBR Green and the standard LightCycler protocol (Roche Diagnostics, Mannheim, Germany). Relative transcript levels calculated from triplicate measurements were calculated by the 2^-ΔΔCT^ method with values normalized to *Gus* and relative to transcription in untreated control cells [63].

### *C-MYB* silencing by siRNA

*C-MYB*-specific siRNA (FlexiTube GeneSolution GS4602 for *c-MYB*) was purchased from Qiagen. As negative controls the same cells were transfected with AllStars Negative Control siRNA (Qiagen), a nonsilencing siRNA with no homology to any known mammalian gene. Transfection was accomplished using the Nucleofector manual (program T016, Lonza GmbH, Köln, Germany). For siRNA treatment, 1.5x10^6^ cells were resuspended in 100 μl Cell Line Nucleofector Solution V (Lonza) containing 18 μl Supplement S Solution (Lonza). The siRNA was added to a final concentration of 0.01 nmol per 10^6^ cells. 24 h after transfection *c-MYB* transcript levels were analyzed by qRT-PCR. Protein lysates for Western blot immunostaining experiments were prepared 48 h after transfection.

### *ESPL1* promoter electrophoretic mobility shift assay (EMSA)

Native nuclear extracts were prepared from BV-173 cells according to a rapid micropreparation method [64]. FITC-labeled oligonucleotides (32mers) corresponding to the c-MYB-binding site sequence in the human *ESPL1* promoter (Human genome browser http://www.ncbi.nlm.nih.gov/gene?cmd=Retrieve&dopt=Graphics&list_uids=9700), NC_000012.11 chromosome 12 reference GRCh37.p13 primary assembly; c-MYB binding position: chr12: 53,646,460 - 53,646,470) were synthesized by Sigma GmbH (Rödermark, Germany). Oligonucleotide sequences (c-MYB binding sequence underlined) were: MYB_sense, FITC-CTCCCACCCACCCAACTGGTCCCTCCGGTCTG; MYB_antisense, FITC-CAGACCGGAGGGACCAGTTGGGTGGGTGGGAG. EMSA was performed using the EMSA kit (order no. E33075) of Life Technologies GmbH (Darmstadt, Germany) according to the instructions of the manufacturer. In brief, 2 μg of nuclear extract were mixed with binding buffer prior to addition of 30 ng of annealed double stranded oligonucleotide corresponding to the c-MYB binding site within the *ESPL1* promoter. After incubation for 20 min at RT, 2 μl of EMSA loading dye was added and the DNA/protein complexes were resolved by electrophoresis (80 V for 90 min) on a 0.5 % TBE 1.0 % native LE/GTG agarose gel. Detection of FITC-labeled DNA oligonucleotides was performed using a ChemiDoc™ XRS+ System (BIO-RAD). Consecutively, the same agarose gel was blotted onto Immobilon-P membrane (Millipore, Bedford, USA) using a Trans Blot SD semi-dry electrophoretic transfer cell (BIO-RAD) at 15 V (current limit at 5 mA/cm^2^) for 30 min. C-MYB Protein detection was performed as described in the Western blot analysis section.

### Chromatin immunoprecipitation (ChIP)

ChIP was performed using the EpiTect ChIP OneDay Kit (#334471, Qiagen, Hilden) according to the instructions of the manufacturer. In brief, 1.5x10^6^ BV-173 cells per sample were lysed and sonicated (5 times 6 s, 0.5 W) using the Vibra-Cell™ system (Sonics, Newtown, USA). Immunoprecipitation was performed with an anti-c-MYB antibody (ab17851, Abcam, Cambridge, UK) and an unspecific control IgG1 control antibody (ab91353, Abcam) according to the manual. For quantification of the immunoprecipitated DNA target, the ChIP qPCR Primer Assay GPH1003144(+)02A (Sabioscience/Qiagen) was applied employing the Roche LightCycler 480 System, using LC480 DNA Master SYBR Green and the standard LightCycler protocol as already described (Roche Diagnostics, Mannheim, Germany). Results derived from triplicate experiments were expressed as percentage of input sample taken before immunoprecipitation during the ChIP procedure. Therefore, the Ct values of the IP fractions were normalized to the input fraction.

### Statistical analysis

Statistical significance of unpaired data was analyzed by the Student’s *t*-test using the GraphPad Prism software version 6.0 (GraphPad Inc., La Jolla, USA). Values of *p* < 0.05 were considered significant.

## Abbreviations

AP: accelerated phase
BC: blast crisis
ChIP: chromatin immunoprecipitation
CML: chronic myeloid leukemia
CP: chronic phase
EMSA: electrophoretic mobility shift assay
ERE: estrogen responsive element
IM: imatinib
PRE: progesterone responsive element
qRT-PCR: quantitative reverse transcription-PCR
RNAi: RNA-interference
TK: tyrosine kinase
TSS: transcription start site
